# Power Resource Optimization for Backscatter-Aided Symbiotic Full-Duplex Secondary Transmission with Hardware Impairments in a Cognitive Radio Framework

**DOI:** 10.3390/s22010375

**Published:** 2022-01-04

**Authors:** Derek Kwaku Pobi Asiedu, Ji-Hoon Yun

**Affiliations:** Department of Electrical and Information Engineering, Seoul National University of Science and Technology, Seoul 01811, Korea; kwakupobi@gmail.com

**Keywords:** spectrum sharing, backscatter communication, full-duplex system, resource allocation

## Abstract

This paper investigates the power resource optimization problem for a new cognitive radio framework with a symbiotic backscatter-aided full-duplex secondary link under imperfect interference cancellation and other hardware impairments. The problem is formulated using two approaches, namely, maximization of the sum rate and maximization of the primary link rate, subject to rate constraints on the secondary link, and the solution for each approach is derived. The problem of a half-duplex secondary link is also solved. Simulation results show that the sum rate and exploitation of the full-duplex capability of the secondary link are strongly affected by both the problem objective and hardware impairments.

## 1. Introduction

Cognitive radio (CR) is shifting spectrum usage from fixed allocation to the sharing/exploration of new spectrum resources [1]. Spectrum resources are efficiently used in the CR framework (CRF) by employing spectrum sensing or power allocation [2,3,4]. Backscatter communication (BC) technology, in which devices transmit data by backscattering waves from radio transmitters, has also emerged to support the realization of ultra-low-power and ultra-low-cost devices [5,6,7,8]. Recently, attempts have been made to combine CRF and BC technology in two different approaches: (1) BC-based secondary transmission [9,10,11,12,13] and (2) BC-aided secondary transmission [14,15,16,17]. In particular, the BC-aided approach improves the performance of not only secondary transmission but also primary transmission if the power resources are properly allocated; thus, this approach is also referred to as the *symbiotic radio* approach [1] due to the diversity gain achieved through the additional communication path using a BC device.

Most studies on BC-aided secondary transmission have focused on a half-duplex (HD) secondary link (SL) [14,16,17,18]. With the advent of single-channel, full-duplex (FD) communication technology, FD- and BC-based SLs have been evaluated in several research works [11,13]. However, FD-based, BC-aided SLs have not yet been explored in the literature. Since the adoption of FD communication in combination with a BC-aided SL prompted the creation of new interference scenarios, this new type of system needs exploration. Currently, it is known that imperfect self-interference cancellation (SIC) and other hardware impairments (HIs) [19,20] greatly affect the performance of FD communication, and it is important to consider them in resource allocation [11,13,21,22].

Since FD-based, BC-aided, SL transmission has not been considered in existing BC-aided transmission research, the effects and influence of hardware impairments, including imperfect SIC, have not been studied either in the CRF with FD-based, BC-aided, and SL transmission. Moreover, communicating devices have other imperfections within their hardware [19], which are introduced in devices due to amplifier non-linearity, in-phase/quadrature imbalance, quantization error, etc. [20]. Hence, HIs must be considered within the CRF with BC-aided transmission. However, HIs have not been explored in the CRF with BC-aided transmission [14,16,18]. In addition to HIs, power resources must be efficiently allocated to improve the CRF performance. However, most BC-aided transmission research (e.g., [16]) has focused on a system analysis. In [14], the authors considered the power resource allocation optimization of BC-aided transmission; however, this is HD without HI. Therefore, the power resource optimization problem for FD-based, BC-aided transmission in the CRF with HIs needs to be investigated.

In this paper, we solve the power resource optimization problem for the new CRF with a symbiotic, FD-based, BC-aided SL protecting a primary link (PL), considering HIs for the nodes of both links. In the proposed CRF, the SL consists of two FD nodes that simultaneously transmit to each other. On both the PL and SL, information is sent via a direct link and a BC-aided tag link. We consider various HI cases: imperfect SIC (Imp-SIC), imperfect successive interference cancellation (Imp-SuIC), and other HIs causing signal distortion and channel estimation error. The power resource optimization problem is solved using two approaches with different objectives: (a) maximizing the sum rate (MSR) and (b) maximizing the PL rate subject to rate constraints on the SL (MR). The solution for the FD mode is obtained with a simple root-finding algorithm, while that for the HD mode is derived in a closed form. Through simulation, we show that the sum rate and the exploitation of the FD capability of the SL are strongly affected by both the problem objective and HIs.

The remainder of the paper is organized as follows. Recent studies related to this work are reviewed and discussed in Section 2. The system model under consideration is introduced in Section 3. Section 4 presents an optimization solution with FD transmission, and Section 5 presents it with HD transmission. Section 6 presents the performance evaluation, and Section 7 concludes the paper.

*Notations: *E[X] is the expectation of the random variable *X*. $n_{a}∼\mathrm{CN}\left(0,\sigma_{a}^{2}\right)$ defines a circularly additive white Gaussian noise (AWGN) variable $n_{a}$ with a mean of zero and a variance of $\sigma_{a}^{2}$.

## 2. Related Work

In this section, the related works focus on BC, unidirectional (one-way) and bidirectional (two-way) FD and HD communication systems. A time-sharing HD CRF between PL transmission and BC-aided SL transmission is presented in [23]. The authors proposed time and power allocation schemes to optimize the transmission rates for the unidirectional, HD, CRF system. Another unidirectional, BC-aided, SL transmission is considered in [16]. The work focused on analyzing the influence of the SL signal interference in the PL communication. The authors in [24] focused on analyzing the capacities of an HD, unidirectional system consisting of a combination of SL, BC-transmission and BC-aided, PL transmission in their research. A unidirectional, SL, BC-transmission system is discussed in [25]. The authors considered both the equal symbol period and unequal symbol period between the primary transmitter (PT) and the secondary transmitter (ST) and maximized the system sum rate. Outage analysis on a unidirectional BC-aided PL with energy harvesting SL are presented in [9,26].

In [27], the authors presented an HD two-way BC-aided PL communication, where the two SL, BC transmitters aided two PL devices to communicate with each other. The two SL transmitters also communicate with each other. Communication between the inter-link devices occurs using HD and time division multiple access (TDMA). The authors performed outage analysis on their proposed system model. Another bidirectional BC communication system is analyzed in [17] where a hybrid relay (BC transmitter and data relay) aids communication between two PL devices. The hybrid relay acts as a BC-aided transmission device for the two PL devices and acts as a BC-transmission device to achieve BC between its data and the two PL devices. An energy harvesting TDMA HD bi-directional BC-transmission communication system is investigated in [28]. In their system model, the access point (AP) transmits energy and data in two different time slots and receives backscattered data and conventional transmission data from sensors within the network topology.

An FD, unidirectional BC-aided PL transmission is presented in [29]. The authors considered a hybrid, FD device that decodes information and backscatters (BC-aided transmission) signals to the primary receiver. This process was conducted to improve the spectral efficiency of the primary receiver. Another FD consideration where bidirectional communication occurs for SL BC-transmission is discussed in [11]. In [11], an FD AP acts as the primary transmitter and secondary receiver, which transmits and collects data using TDMA. The focus of this paper was to maximize the system rate based on time resource optimization. An FD SL BC-transmission system is presented in [30]. In [30], the FD, secondary AP transmits energy signals and receives BC data transmission from the secondary users. Both the PL and SL cause interference with each other. The authors seek to maximize the CRF sum rate.

As evident by the related works discussed, most papers concentrate on HD and unidirectional BC-(transmission-aided) PL and SL communications [9,16,23,24,25,26]. Fewer of these studies focus on unidirectional FD-based BC PL, and SL communication [11,29,30]. Concerning bidirectional BC PL, and SL communication, [17,27,28] considered BC-aided (transmission-aided) HD, PL, and SL communication. Research on bidirectional FD BC-aided and BC-transmission communication have not been considered in research.

## 3. System Model

**Figure 1:**
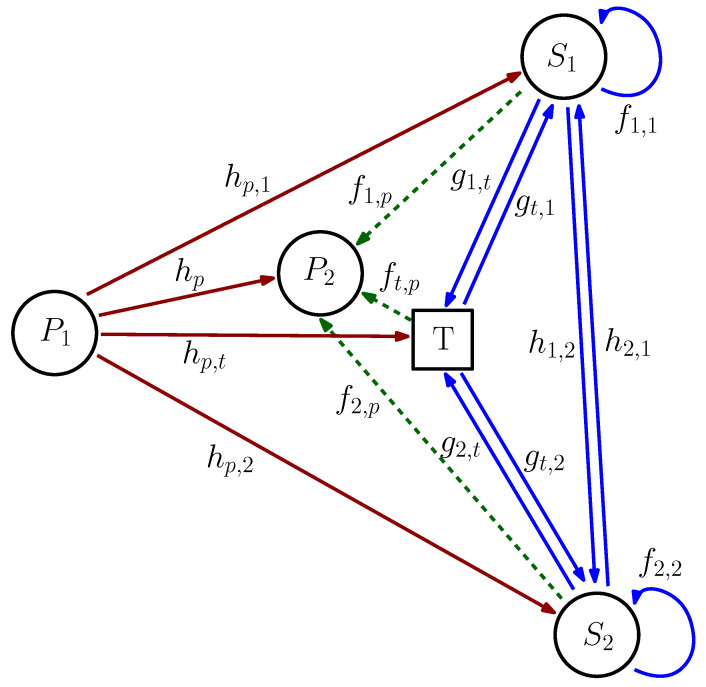
System layout of the symbiotic BC-aided CRF with an FD-based SL.

A BC-aided CRF consisting of a PL and an FD-based SL sharing a spectrum band is illustrated in Figure 1. The PL consists of a transmitter $P_{1}$ and a receiver $P_{2}$. The SL consists of two FD-capable STs $S_{1}$ and $S_{2}$ that communicate with each other. Both links transfer information over a direct link and a BC link facilitated by a BC tag *T*. The tag BC link helps improve the spectral efficiency of both the PL and SL due to improvement in diversity gain [1]. Unlike [16], we consider non-negligible interference with the SL introduced by $P_{1}$ for a more realistic deployment scenario. $P_{2}$ suffers interference from the nearby STs and tag. Imp-SIC (for the cancellation of self-interference) and Imp-SuIC (for the cancellation of the interference from $P_{1}$) are considered for $S_{1}$ and $S_{2}$, while the other HIs resulting in signal distortion and channel estimation error are considered for all nodes, including $P_{1}$ and $P_{2}$. The internode channels are depicted and defined in Figure 1. Channel reciprocity is assumed since all communication paths are established in the same frequency band. $P_{P_{1}}$, $P_{S_{1}}$, and $P_{S_{2}}$ are the transmit power levels of $P_{1}$, $S_{1}$, and $S_{2}$, respectively, and ${\bar{P}}_{P_{1}}$, ${\bar{P}}_{S_{1}}$, and ${\bar{P}}_{S_{2}}$ are their maximum values. The transmitted signal $x_{n}$ is assumed to satisfy $E[|x_{n}{|}^{2}]=1$ ($n\in \{P_{1},S_{1},S_{2},t\}$, where *t* represents the tag *T*). η (0<η≤1) is the BC signal attenuation factor.

The received signal at $P_{2}$ is obtained as

(1) $$\begin{array}{cc} \; & \;\;y_{P_{2}}=\underset{\mathrm{Desired}\;P_{1}\mathrm{-backscattered}\;\mathrm{link}\;+\;\mathrm{direct}\;\mathrm{link}\;\mathrm{signals}}{\underset{︸}{\left(h_{p}+\sqrt{\eta }h_{p,t}f_{t,p}x_{t}\right)\left(\sqrt{P_{P_{1}}}x_{P_{1}}+n_{P_{1},P_{2}}\right)}}+\phi_{P_{2}}+\underset{S_{1}\mathrm{-backscattered}\;\mathrm{link}\;+\;\mathrm{direct}\;\mathrm{link}\;\mathrm{interference}\;\mathrm{signals}}{\underset{︸}{\left(f_{1,p}+\sqrt{\eta }g_{1,t}f_{t,p}x_{t}\right)\left(\sqrt{P_{S_{1}}}x_{S_{1}}+n_{S_{1},P_{2}}\right)}}\\ & \;+\underset{S_{2}\mathrm{-backscattered}\;\mathrm{link}\;+\;\mathrm{direct}\;\mathrm{link}\;\mathrm{interference}\;\mathrm{signals}}{\underset{︸}{\left(f_{2,p}+\sqrt{\eta }g_{2,t}f_{t,p}x_{t}\right)\left(\sqrt{P_{S_{2}}}x_{S_{2}}+n_{S_{2},P_{2}}\right)}}, \end{array}$$

where $n_{z_{1},z_{2}}∼CN\left(0,\xi_{z_{1},z_{2}}^{2}P_{z_{1}}\right)$ is the distortion noise of a received signal on a directional link from node $z_{1}$ to node $z_{2}$ (where $z_{1}$ and $z_{2}$ may represent $P_{1}$, $P_{2}$, $S_{1}$, or $S_{2}$), the variance of which is defined as $\xi_{z_{1},z_{2}}^{2}=\sqrt{\xi_{z_{1}}^{2}+\xi_{z_{2}}^{2}}$. The antenna noise at node *z* ($P_{2}$, $S_{1}$ or $S_{2}$) is defined as $\phi_{z}∼\mathrm{CN}\left(0,\sigma_{z}^{2}\right)$. The received signals at the STs are given by

(2) $$\begin{array}{cc} \; & \;\;y_{S_{i}}=\underset{\mathrm{Desired}\;S_{j}\mathrm{-backscattered}\;\mathrm{link}\;+\;\mathrm{direct}\;\mathrm{link}\;\mathrm{signals}}{\underset{︸}{\left(h_{j,i}+\sqrt{\eta }g_{j,t}g_{t,i}x_{t}\right)\left(\sqrt{P_{S_{j}}}x_{S_{j}}+n_{S_{j},S_{i}}\right)x_{t}}}+\phi_{S_{i}}+\underset{S_{i}\mathrm{-backscattered}\;+\;\mathrm{self-interference}\;\mathrm{signals}}{\underset{︸}{\left(f_{i,i}+\sqrt{\eta }g_{i,t}g_{t,i}x_{t}\right)\left(\sqrt{P_{S_{i}}}\left(x_{S_{i}}-{\hat{x}}_{S_{i}}\right)+n_{S_{i}}\right)}}\\ & \;+\underset{P_{1}\mathrm{-backscattered}\;\mathrm{link}\;+\;\mathrm{direct}\;\mathrm{link}\;\mathrm{interference}\;\mathrm{signals}}{\underset{︸}{\left(h_{p,i}+\sqrt{\eta }h_{p,t}g_{t,i}x_{t}\right)\left(\sqrt{P_{P_{1}}}x_{P_{1}}+n_{P_{1},S_{i}}\right)}}, \end{array}$$

where (i,j)∈{(1,2),(2,1)}; $n_{z}∼CN\left(0,\xi_{z}^{2}P_{z}\right)$ is the distortion noise of a self-interference signal present at node *z* ($S_{1}$ or $S_{2}$), with variance $\xi_{z}^{2}$, and ${\hat{x}}_{S_{i}}$ is the estimated signal of $S_{i}$.

## 4. Backscatter-Aided CR Framework with FD Secondary Transmission under HIs

The interference power at $P_{2}$ is defined as

(3) $$Q_{s}=P_{S_{1}}\left(\right|f_{1,p}{|}^{2}+\eta |g_{1,t}{|}^{2}|f_{t,p}{|}^{2})\left(1+\xi_{S_{1},P_{2}}^{2}\right)+P_{S_{2}}\left(\right|f_{2,p}{|}^{2}+\eta |g_{2,t}{|}^{2}|f_{t,p}{|}^{2})\left(1+\xi_{S_{2},P_{2}}^{2}\right).$$

We introduce the control variable β (0≤β≤1) to adjust the interference power components of $S_{1}$ and $S_{2}$ to $\beta Q_{s}$ and $\left(1-\beta \right)Q_{s}$, respectively, by means of a resource allocation algorithm. Therefore, the transmit power levels of $S_{1}$ and $S_{2}$ are given by

(4) $$P_{S_{1}}=\min\left\{\frac{\beta Q_{s}}{\left(\right|f_{1,p}{|}^{2}+\eta |g_{1,t}{|}^{2}|f_{t,p}{|}^{2})\left(1+\xi_{S_{1},P_{2}}^{2}\right)},{\bar{P}}_{S_{1}}\right\}$$

and

(5) $$P_{S_{2}}=\min\left\{\frac{\left(1-\beta \right)Q_{s}}{\left(\right|f_{2,p}{|}^{2}+\eta |g_{2,t}{|}^{2}|f_{t,p}{|}^{2})\left(1+\xi_{S_{2},P_{2}}^{2}\right)},{\bar{P}}_{S_{2}}\right\},$$

respectively, which are determined by controlling $Q_{s}$ and β. We then obtain the signal-to-interference-plus-noise ratios (SINRs) at $P_{2}$, $S_{1}$, and $S_{2}$ as

(6) $${\hat{\gamma }}_{P_{2}}=\frac{P_{P_{1}}\left(\right|h_{p}{|}^{2}+\eta |h_{p,t}{|}^{2}|f_{t,p}{|}^{2})}{Q_{s}+P_{P_{1}}\left(\right|h_{p}{|}^{2}+\eta |h_{p,t}{|}^{2}|f_{t,p}{|}^{2})\xi_{P_{1},P_{2}}^{2}+\sigma_{p,2}^{2}},$$

(7) $${\hat{\gamma }}_{S_{1}}=\frac{\left(1-\beta \right)Q_{s}B_{1,1}}{\beta Q_{s}B_{2,1}+B_{3,1}+\left(1-\beta \right)Q_{s}B_{4,1}}$$

and

(8) $${\hat{\gamma }}_{S_{2}}=\frac{\beta Q_{s}B_{1,2}}{\left(1-\beta \right)Q_{s}B_{2,2}+B_{3,2}+\beta Q_{s}B_{4,2}},$$

respectively. Here,

(9) $$B_{1,i}=\;|h_{j,i}{|}^{2}+\eta |g_{j,t}{|}^{2}{\left|g_{t,i}\right|}^{2},\;B_{4,i}=B_{1,i}\xi_{S_{j},S_{i}}^{2},$$

(10) $$B_{3,i}=\;[P_{P_{1}}\left(\right|h_{p,i}+\eta |h_{p,t}{|}^{2}|g_{t,i}{|}^{2})\left(\chi_{S_{i}}+\xi_{P_{1},S_{i}}^{2}\right)+\sigma_{S_{i}}^{2}](|f_{j,p}{|}^{2}+\eta |g_{j,t}{|}^{2}|f_{t,p}{|}^{2})\left(1+\xi_{S_{j},P_{2}}^{2}\right),$$

and

(11) $$B_{2,i}=B_{2,i}^{\mathrm{sub}1}/B_{2,i}^{\mathrm{sub}2},$$

where the subs are defined as

(12) $$B_{2,i}^{\mathrm{sub}1}=\left(\right|f_{i,i}{|}^{2}+\eta |g_{i,t}{|}^{2}|g_{t,i}{|}^{2})(|f_{j,p}{|}^{2}+\eta |g_{j,t}{|}^{2}|f_{t,p}{|}^{2})\left(\kappa_{S_{i}}+\xi_{S_{i}}^{2}\right)\left(1+\xi_{S_{j},P_{2}}^{2}\right)$$

and

(13) $$B_{2,i}^{\mathrm{sub}2}=\left(\right|f_{i,p}{|}^{2}+\eta |g_{i,t}{|}^{2}|f_{t,p}{|}^{2})\left(1+\xi_{S_{i},P_{2}}^{2}\right).$$

$\kappa_{S_{i}}$ and $\chi_{S_{i}}$ are the Imp-SIC coefficient and Imp-SuIC coefficient, respectively, at $S_{i}$. A larger coefficient value means worse cancellation performance and higher residual interference. The rates achieved at $P_{2}$, $S_{1}$ and $S_{2}$ are given by

(14) $${\hat{R}}_{P_{2}}=BW\log_{2}\left(1+{\hat{\gamma }}_{P_{2}}\right),\;{\hat{R}}_{S_{1}}=BW\log_{2}\left(1+{\hat{\gamma }}_{S_{1}}\right),\;\mathrm{and}\;{\hat{R}}_{S_{2}}=BW\log_{2}\left(1+{\hat{\gamma }}_{S_{2}}\right),$$

respectively, where BW is the bandwidth.

Two power resource optimization problems for the system are considered as follows (Note that the focus of the optimization problems considered in this paper is to reduce (minimize) the interference of the SL within the PL. Hence, the PL transmit power is not considered a variable in the optimization problems.).

### 4.1. Sum-Rate Maximization (MSR)

The system sum rate is maximized by optimizing the power resources of the SL, specifically, $Q_{s}$ and β. The problem corresponding to the MSR approach is expressed as

(15) $$\begin{array}{cc} \; & \underset{Q_{s},\beta }{\mathrm{maximize}}\;{\hat{R}}_{P_{2}}+{\hat{R}}_{S_{1}}+{\hat{R}}_{S_{2}}\;\mathrm{subject}\;\mathrm{to}\;Q_{s}\geq 0,\;0\leq \beta \leq 1. \end{array}$$

The solution to the MSR problem is presented in Theorem 1 below, and the proof is given here.

**Theorem** **1.** *The optimum for the MSR problem is obtained at*

$$Q_{s}^{★}=\frac{B_{3,1}{\hat{a}}_{2}^{★}+B_{3,2}\left({\hat{a}}_{3}^{★}+B_{2,1}\right)}{{\hat{a}}_{2}^{★}{\hat{a}}_{3}^{★}-B_{3,2}\left({\hat{a}}_{3}^{★}+B_{2,1}\right)}$$

*and*

$$\beta^{★}=\frac{B_{3,1}B_{2,2}+B_{3,2}{\hat{a}}_{3}^{★}}{B_{3,1}{\hat{a}}_{2}^{★}+B_{3,2}\left({\hat{a}}_{3}^{★}+B_{2,1}\right)},$$

*where ${\hat{a}}_{2}^{★}$ and ${\hat{a}}_{3}^{★}$ are the roots of the quadratic equation*

$$B_{1,1}\left({\hat{a}}_{3}^{★}+B_{2,1}\right)\left(B_{3,1}^{2}+{\hat{a}}_{3}^{★}B_{3,1}\left(1-\lambda \right)+{\hat{a}}_{3}^{★}B_{3,1}\lambda \right)\left({\hat{a}}_{2}^{★}-B_{2,2}+B_{4,2}\right)\left(B_{1,2}+{\hat{a}}_{2}^{★}-B_{2,2}+B_{4,2}\right)$$

$$-{\hat{a}}_{2}^{★}B_{1,2}\left({\hat{a}}_{2}^{★}B_{3,1}-B_{3,1}^{2}+B_{2,1}B_{3,2}\left(1-\lambda \right)+B_{2,1}B_{3,1}\lambda \right)\left({\hat{a}}_{3}^{★}+B_{4,1}\right)\left(B_{1,1}+{\hat{a}}_{3}^{★}+B_{4,1}\right)=0.$$

**Proof.**  The MSR problem given in (15) is non-convex with respect to all variables. The second derivative approach can be used to check convexity. New variables $Q_{s}$ and β are introduced to help solve the problem. First, we rearrange the SINRs of $S_{1}$ and $S_{2}$ as

$$\frac{B_{1,1}}{\frac{\beta Q_{s}B_{2,1}+B_{3,1}+\left(1-\beta \right)Q_{s}B_{4,1}}{\left(1-\beta \right)Q_{s}}}\;\mathrm{and}\;\frac{B_{1,2}}{\frac{\left(1-\beta \right)Q_{s}B_{2,2}+B_{3,2}+\beta Q_{s}B_{4,2}}{\beta Q_{s}}},$$

respectively. We define

$$a_{1}=\frac{\beta Q_{s}B_{2,1}+B_{3,1}+\left(1-\beta \right)Q_{s}B_{4,1}}{\left(1-\beta \right)Q_{s}}\;\mathrm{and}\;a_{2}=\frac{\left(1-\beta \right)Q_{s}B_{2,2}+B_{3,2}+\beta Q_{s}B_{4,2}}{\beta Q_{s}}.$$

Setting β and $Q_{s}$ on the left-hand sides of the equations for $a_{1}$ and $a_{2}$ results in

$$\beta =\frac{B_{3,1}B_{2,2}+B_{3,2}\left(a_{1}-B_{4,1}\right)}{B_{3,1}\left(a_{2}+B_{2,2}-B_{4,2}\right)+B_{3,2}\left(a_{1}+B_{2,1}-B_{4,1}\right)}$$

and

$$Q_{s}=\frac{B_{3,1}\left(a_{2}+B_{2,2}-B_{4,2}\right)+B_{3,2}\left(a_{1}+B_{2,1}-B_{4,1}\right)}{\left(a_{1}-B_{4,1}\right)\left(a_{2}+B_{2,2}-B_{4,2}\right)-B_{2,2}\left(a_{1}+B_{2,1}-B_{4,1}\right)}.$$

Now, let ${\hat{a}}_{2}=a_{2}+B_{2,2}-B_{4,2}$ and ${\hat{a}}_{3}=a_{1}-B_{4,1}$. Substituting ${\hat{a}}_{2}$ and ${\hat{a}}_{3}$ into the expressions for β and $Q_{s}$ yields

$$\beta =\frac{B_{3,1}B_{2,2}+B_{3,2}{\hat{a}}_{3}}{B_{3,1}{\hat{a}}_{2}+B_{3,2}\left({\hat{a}}_{3}+B_{2,1}\right)}\;\mathrm{and}\;Q_{s}=\frac{B_{3,1}{\hat{a}}_{2}+B_{3,2}\left({\hat{a}}_{3}+B_{2,1}\right)}{{\hat{a}}_{3}{\hat{a}}_{2}-B_{2,2}\left({\hat{a}}_{3}+B_{2,1}\right)}.$$

We then substitute the expressions for $a_{1}$, $a_{2}$, β, and $Q_{s}$ into the objective function of Problem (15) and differentiate it with respect to ${\hat{a}}_{2}$ and ${\hat{a}}_{3}$. We also set the two resulting differentials equal to zero and solve them. The following quadratic problem is obtained

$$B_{1,1}\left({\hat{a}}_{3}^{★}+B_{2,1}\right)\left(B_{3,1}^{2}+{\hat{a}}_{3}^{★}B_{3,1}\left(B_{3,2}-B_{2,2}\lambda \right)+{\hat{a}}_{3}^{★}B_{3,1}\lambda \right)\left({\hat{a}}_{2}^{★}-B_{2,2}+B_{4,2}\right)(B_{1,2}+{\hat{a}}_{2}^{★}$$

$$-B_{2,2}+B_{4,2})-{\hat{a}}_{2}^{★}B_{1,2}\left({\hat{a}}_{2}^{★}B_{3,1}-B_{3,1}^{2}+B_{2,1}B_{3,2}\left(B_{3,2}-B_{2,2}\lambda \right)+B_{2,1}B_{3,1}\lambda \right)\left({\hat{a}}_{3}^{★}+B_{4,1}\right)\left(B_{1,1}+{\hat{a}}_{3}^{★}+B_{4,1}\right)=0,$$

where

$$\lambda =P_{P_{1}}\left(\right|h_{p}{|}^{2}+\eta |h_{p,t}{|}^{2}|f_{t,p}{|}^{2})\xi_{P_{1},P_{2}}^{2}+\sigma_{p,2}^{2}.$$

${\hat{a}}_{2}^{★}$ and ${\hat{a}}_{3}^{★}$ can be obtained using a root-finding method. Substituting ${\hat{a}}_{2}^{★}$ and ${\hat{a}}_{3}^{★}$ into the β and $Q_{s}$ expressions yields $\beta^{★}$ and $Q_{s}^{★}$. □

**Remark** **1.** *The MSR approach does not promote any prioritization with a performance guarantee between the PL and the SL. For example, $Q_{s}$ may be reduced to improve/increase the PL rate, minimizing the rate achieved by the SL. Therefore, maximizing the sum rate may produce meager rates on either the PL or SL. To promote some level of prioritization and a minimal performance guarantee, the PL rate can be maximized while imposing a quality-of-service (QoS) constraint on the SL to achieve at least a minimum desirable rate; this finding is considered in the next approach.*

### 4.2. PL-Rate Maximization (MR)

This problem is expressed as

(16) $$\begin{array}{cc} \; & \;\;\underset{Q_{s},\beta }{\mathrm{maximize}}\;{\hat{R}}_{P_{2}}\;\mathrm{subject}\;\mathrm{to}\;Q_{s}\geq 0,\;0\leq \beta \leq 1,\;{\hat{R}}_{S_{1}}\geq {\bar{R}}_{S_{1},TH},\;{\hat{R}}_{S_{2}}\geq {\bar{R}}_{S_{2},TH}, \end{array}$$

where only the rate on the PL is maximized, subject to new constraints on the minimum rate thresholds for $S_{1}$ and $S_{2}$. (The primary rate maximization problem has an SL rate constraint to require the SL rate in information detection and to achieve a minimum interference level [1].) The solution to the problem corresponding to the MR approach is presented in Theorem 2; its proof is given here.

**Theorem** **2.** *The optimum for the MR problem is obtained at $Q_{s}^{★}$ and $\beta^{★}$, which are derived as*

$$Q_{s}^{★}=\frac{{\bar{\gamma }}_{S_{2}}B_{3,2}q_{1,1}+{\bar{\gamma }}_{S_{1}}B_{3,1}q_{1,2}}{\left(B_{1,1}-{\bar{\gamma }}_{S_{1}}B_{4,1}\right)q_{1,2}-{\bar{\gamma }}_{S_{2}}B_{2,2}q_{1,1}},\;and\;\beta^{★}=\frac{Q_{s}^{★}\left(B_{1,1}-{\bar{\gamma }}_{S_{1}}B_{4,1}\right)-{\bar{\gamma }}_{S_{1}}B_{3,1}}{Q_{s}^{★}\left({\bar{\gamma }}_{S_{1}}\left(B_{2,1}-B_{4,1}\right)+B_{1,1}\right)},$$

*respectively, with*

$$q_{1,1}=\left({\bar{\gamma }}_{S_{1}}\left(B_{2,1}-B_{4,1}\right)+B_{1,1}\right)\;and\;q_{1,2}=\left(B_{1,2}-{\bar{\gamma }}_{S_{2}}\left(B_{4,2}-B_{2,2}\right)\right),$$

*where ${\bar{\gamma }}_{S_{1}}$ and ${\bar{\gamma }}_{S_{2}}$ are the SINR thresholds of $S_{1}$ and $S_{2}$, respectively.*

**Proof.**  In the MR problem given in (16), the maximum $P_{2}$ rate is achieved under the equality condition of the SL rate constraints, where the SL introduces the lowest $Q_{s}$ at $P_{2}$. Using

$$\frac{\left(1-\beta \right)Q_{s}B_{1,1}}{\beta Q_{s}B_{2,1}+B_{3,1}+\left(1-\beta \right)Q_{s}B_{4,1}}=2^{{\bar{R}}_{S_{1},TH}}-1={\bar{\gamma }}_{S_{1}}$$

and

$$\frac{\beta Q_{s}B_{1,2}}{\left(1-\beta \right)Q_{s}B_{2,2}+B_{3,2}+\beta Q_{s}B_{4,2}}=2^{{\bar{R}}_{S_{2},TH}}-1={\bar{\gamma }}_{S_{2}},$$

the optimal $Q_{s}$ and β are determined to be

$$Q_{s}^{★}=\frac{{\bar{\gamma }}_{S_{2}}B_{3,2}q_{1,1}+{\bar{\gamma }}_{S_{1}}B_{3,1}q_{1,2}}{\left(B_{1,1}-{\bar{\gamma }}_{S_{1}}B_{4,1}\right)q_{1,2}-{\bar{\gamma }}_{S_{2}}B_{2,2}q_{1,1}}\;\mathrm{and}\;\beta^{★}=\frac{Q_{s}^{★}\left(B_{1,1}-{\bar{\gamma }}_{S_{1}}B_{4,1}\right)-{\bar{\gamma }}_{S_{1}}B_{3,1}}{Q_{s}^{★}\left({\bar{\gamma }}_{S_{1}}\left(B_{2,1}-B_{4,1}\right)+B_{1,1}\right)},$$

respectively, where

$$q_{1,1}=\left({\bar{\gamma }}_{S_{1}}\left(B_{2,1}-B_{4,1}\right)+B_{1,1}\right)\;\mathrm{and}\;q_{1,2}=\left(B_{1,2}-{\bar{\gamma }}_{S_{2}}\left(B_{4,2}-B_{2,2}\right)\right).$$

${\bar{\gamma }}_{S_{1}}$ and ${\bar{\gamma }}_{S_{2}}$ are the SL’s SINR thresholds. □

## 5. Backscatter-Aided CRF with HD Secondary Transmission

In the HD operation of the SL, we assume that the STs perform two-way data transmission on a time division basis, while the PL continues transmitting data throughout the whole communication time. The rates for $P_{2}$, $S_{1}$, and $S_{2}$ are given by

$${\hat{R}}_{P_{2}}=\alpha \tau \log_{2}\left(1+\frac{P_{P_{1}}\left(\right|h_{p}{|}^{2}+\eta |h_{p,t}{|}^{2}|f_{t,p}{|}^{2})}{Q_{S_{2}}+P_{P_{1}}\left(\right|h_{p}{|}^{2}+\eta |h_{p,t}{|}^{2}|f_{t,p}{|}^{2})\xi_{P_{1},P_{2}}^{2}+\sigma_{p,2}^{2}}\right)$$

$$+\left(1-\alpha \right)\tau \log_{2}\left(1+\frac{P_{P_{1}}\left(\right|h_{p}{|}^{2}+\eta |h_{p,t}{|}^{2}|f_{t,p}{|}^{2})}{Q_{S_{1}}+P_{P_{1}}\left(\right|h_{p}{|}^{2}+\eta |h_{p,t}{|}^{2}|f_{t,p}{|}^{2})\xi_{P_{1},P_{2}}^{2}+\sigma_{p,2}^{2}}\right),$$

$${\hat{R}}_{S_{1}}=\alpha \tau \log_{2}\left(1+\frac{Q_{S_{2}}B_{1,1}}{B_{3,1}+Q_{S_{2}}B_{4,1}}\right),$$

and

$${\hat{R}}_{S_{2}}=\left(1-\alpha \right)\tau \log_{2}\left(1+\frac{Q_{S_{1}}B_{1,2}}{B_{3,2}+Q_{S_{1}}B_{4,2}}\right),$$

where α (0<α<1) is the time allocation variable for $S_{1}$ (the portion of the time allocated for $S_{2}$ is 1−α) and τ is the total transmission time.

The solution details of the MSR and MR problems for the HD mode of the SL are presented here. The MSR problem can be split into two subproblems: a subproblem for $S_{1}$ reception (i.e., with $Q_{S_{2}}$ as a variable) and a subproblem for $S_{2}$ reception (i.e., with $Q_{S_{1}}$ as a variable). Both subproblems are convex optimization problems. Hence, the solutions are acquired when the subproblems’ first derivatives are equated with zero, yielding

$$Q_{S_{1}}^{★}=\frac{-b_{1}+\sqrt{b_{1}^{2}-4a_{1}c_{1}}}{2a_{1}}\;\mathrm{and}\;Q_{S_{2}}^{★}=\frac{-b_{2}+\sqrt{b_{2}^{2}-4a_{2}c_{2}}}{2a_{2}},$$

where

$$c_{1}=B_{3,2}\left(P_{P_{1}}\mu B_{3,2}-B_{1,2}\lambda \left(P_{P_{1}}\mu +\lambda \right)\right),$$

$$b_{1}=B_{3,2}\left(P_{P_{1}}\mu \left(B_{1,2}-2B_{4,2}\right)-B_{1,2}\left(P_{P_{1}}\mu +2\lambda \right)\right),$$

$$a_{1}=P_{P_{1}}\mu B_{4,2}\left(B_{1,2}+B_{4,2}\right)-B_{1,2}B_{3,2},$$

$$c_{2}=B_{3,1}\left(P_{P_{1}}\mu B_{3,1}-B_{1,1}\lambda \left(P_{P_{1}}\mu +\lambda \right)\right),$$

$$b_{2}=B_{3,1}\left(P_{P_{1}}\mu \left(B_{1,1}-2B_{4,1}\right)-B_{1,1}\left(P_{P_{1}}\mu +2\lambda \right)\right),$$

$$a_{2}=P_{P_{1}}\mu B_{4,1}\left(B_{1,1}+B_{4,1}\right)-B_{1,1}B_{3,1},$$

and

$$\mu =(|h_{p}{|}^{2}+\eta |h_{p,t}{|}^{2}|f_{t,p}{|}^{2}).$$

Similar to the MR problem in the FD mode, the SINR thresholds are applied to determine the $Q_{S_{i}}$ solutions as

$$Q_{S_{1}}^{★}=\frac{{\bar{\gamma }}_{S_{2},TH}B_{3,2}}{B_{1,2}-{\bar{\gamma }}_{S_{2},TH}B_{4,2}}\;\mathrm{and}\;Q_{S_{2}}^{★}=\frac{{\bar{\gamma }}_{S_{1},TH}B_{3,1}}{B_{1,1}-{\bar{\gamma }}_{S_{1},TH}B_{4,1}},$$

where ${\bar{\gamma }}_{S_{1},TH}$ and ${\bar{\gamma }}_{S_{2},TH}$ are the SINR thresholds of $S_{1}$ and $S_{2}$, respectively.

**Remark** **2.** *We can consider multiple HI scenarios by setting the HI coefficient values accordingly. For the perfect cancellation + no HIs (PSNHI) scenario, all HI coefficients are set to zero. For perfect cancellation + HIs, only the Imp-SIC and Imp-SuIC coefficients are set to zero. For imperfect cancellation + HIs (ISHI), all coefficients are set to non-zero values. $Q_{s}$ and β can be determined by $P_{2}$ since it is within the coverage area of both the PL and SL. Hence, it is assumed that $P_{2}$ collects the channel state information (CSI), determines $Q_{s}$ and β, and broadcasts their values to the STs to use. The solution for the HD mode is given in a closed form, whereas for the FD mode, it is necessary to run an additional root-finding algorithm to obtain the solution. Therefore, the overall computational burden on $P_{2}$ is insignificant.*

## 6. Simulation Results and Discussion

In this section, simulation results comparing the FD and HD modes of the SL are presented. Two HI scenarios (PSNHI and ISHI) are considered in addition to varying channel estimation errors. The node-to-node ($z_{1}$-to-$z_{2}$) channels are modeled as $\sqrt{A_{0}{\left(C_{L}/\left(4\pi f_{c}d_{z_{1},z_{2}}\right)\right)}^{\delta }G_{T}G_{R}}\zeta_{z_{1},z_{2}}$, while the tag-to-node channels are modeled as $d_{z_{1},z_{2}}^{-\delta }\zeta_{z_{1},z_{2}}$ [22] where $d_{z_{1},z_{2}}$ is the internode distance. The small-scale fading is defined as Rayleigh fading, of the form $\zeta_{z_{1},z_{2}}∼\mathrm{CN}\left(0,1\right)$. (Unless specified otherwise, we consider uncorrelated Rayleigh fading channels.)

**Figure 2:**
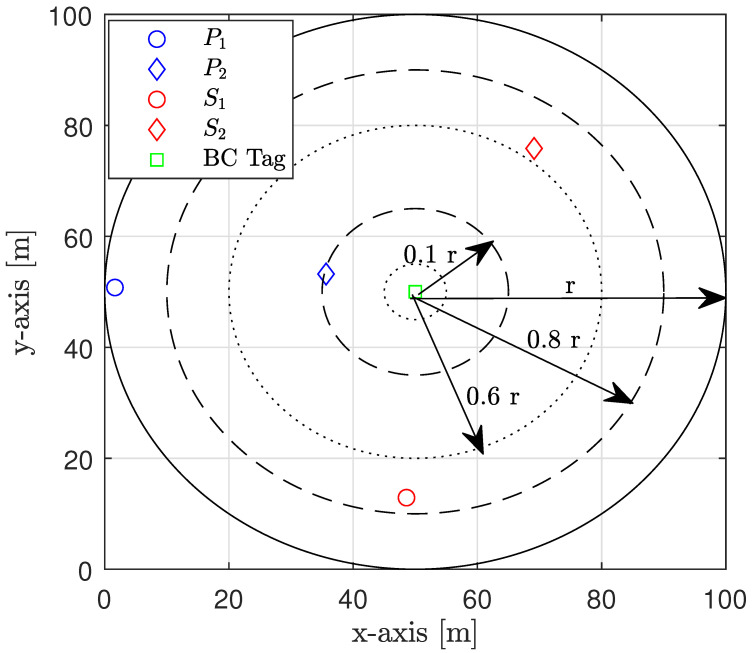
Example of the simulated system topology.

The nodes (i.e., PT, PR, and STs) are randomly distributed within a specified radius from the BC tag, as shown in the example in Figure 2. Unless specified otherwise, the parameter values utilized for simulation are those listed in Table 1. The results presented are achieved over $10^{6}$ random channel generations.

**Figure 3:**
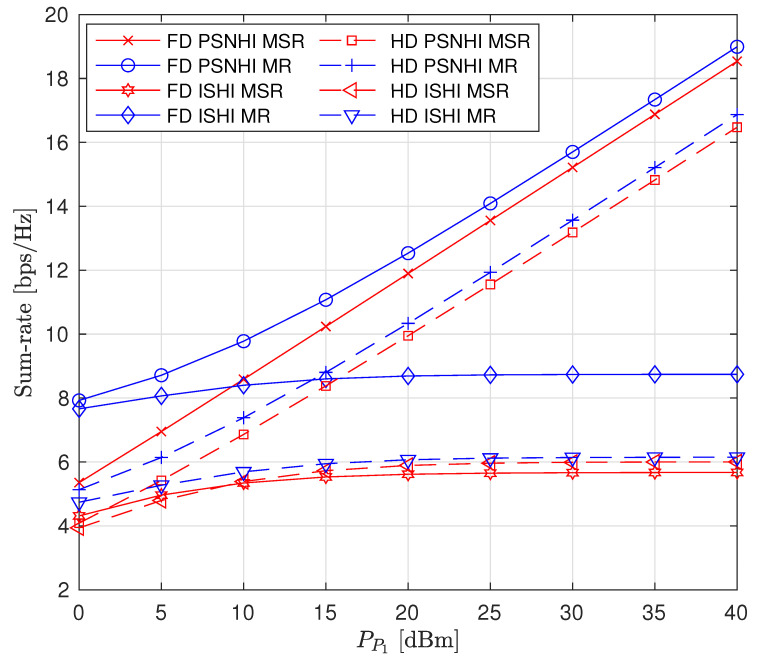
Sum rate versus primary transmitter (PT) transmit power ($P_{P_{1}}$).

**Figure 4:**
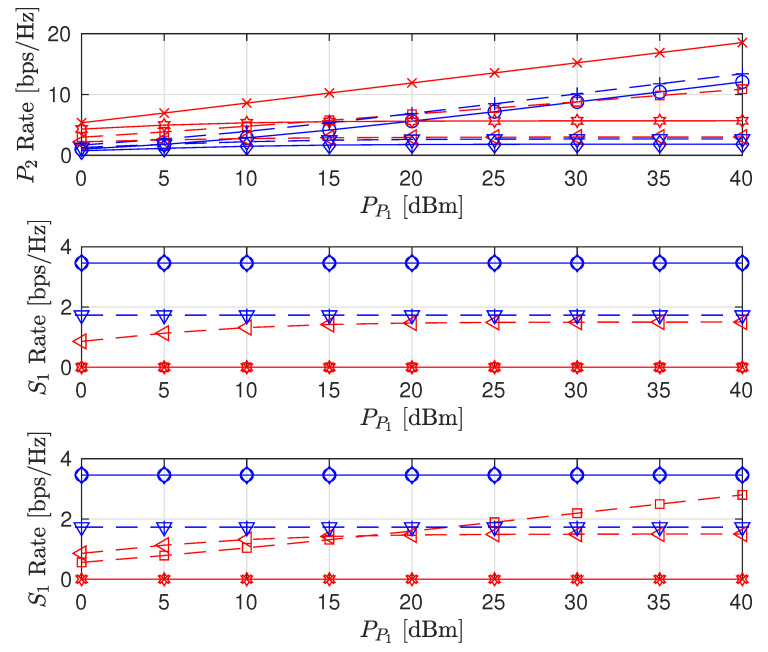
Achievable node rate versus PT transmit power.

**Figure 5:**
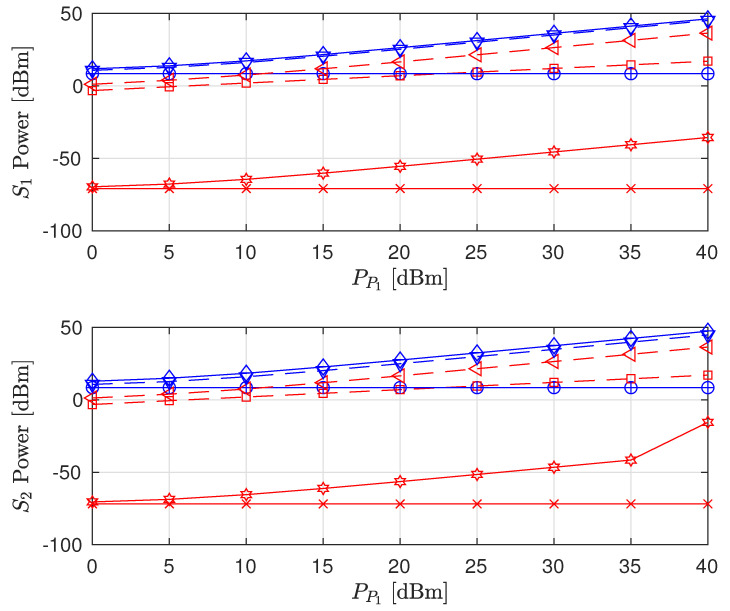
Secondary node transmit power versus PT transmit power.

The effects of increasing $P_{P_{1}}$ on the sum rate (i.e., ${\hat{R}}_{P_{2}}+{\hat{R}}_{S_{1}}+{\hat{R}}_{S_{2}}$), individual node rates and the transmit power levels of the STs are shown in Figure 3, Figure 4 and Figure 5, respectively (The legends are the same for all plots; thus, they are not shown on all plots for graph visibility.). First, we compare the MSR and MR approaches. In Figure 3, the MR approach outperforms the MSR approach for both the PSNHI and ISHI scenarios in terms of the sum rate. This observation can be explained by Figure 4, where it is seen that the STs achieve insignificant rate values under the MSR approach because they are forced to use little power to reduce the interference at $P_{2}$, as shown in Figure 5. In contrast, the MR approach allows the STs to use higher transmit power to satisfy their rate constraints, as shown in Figure 4. Second, we compare the FD and HD modes for the different approaches and HI scenarios. As shown in Figure 3, under the MR approach, the FD mode achieves better sum rates than the HD mode in both considered HI scenarios. This result occurs with similar transmit power levels between the STs for both modes, as shown in Figure 5, although the $S_{1}$ and $S_{2}$ rates are almost doubled in the FD mode due to simultaneous transmission and reception, as observed in Figure 4. However, the MSR approach cannot take advantage of the benefits of the FD mode since the goal of this approach is to minimize the interference from $S_{1}$ and $S_{2}$ at $P_{2}$, thereby maximizing ${\hat{R}}_{P_{2}}$ instead of concurrently maximizing ${\hat{R}}_{S_{1}}$ and ${\hat{R}}_{S_{2}}$. Note that the transmit power of $P_{1}$ is given, while the transmit power levels of $S_{1}$ and $S_{2}$ are determined reactively and thus, are set to be insignificant in the MSR approach. This finding is confirmed by the SL transmit power levels and rates in Figure 4 and Figure 5, respectively. In particular, in the ISHI scenario, the FD mode achieves a lower sum rate than the HD mode because the system imperfections require the STs to use higher transmit power to increase their rates, which increases the interference affecting $P_{2}$. Under the MSR approach in the PSNHI scenario, the FD mode outperforms the HD mode because $P_{2}$ attains a larger rate in the former compared to the latter because the SL interference power is minimal in the FD mode. Notably, sum-rate saturation is observed in Figure 3 for both the FD and HD modes in the ISHI scenario due to an excessive interference-and-noise sum resulting from $P_{1}$’s interference, the residual interference after cancellation and other HIs.

**Figure 6:**
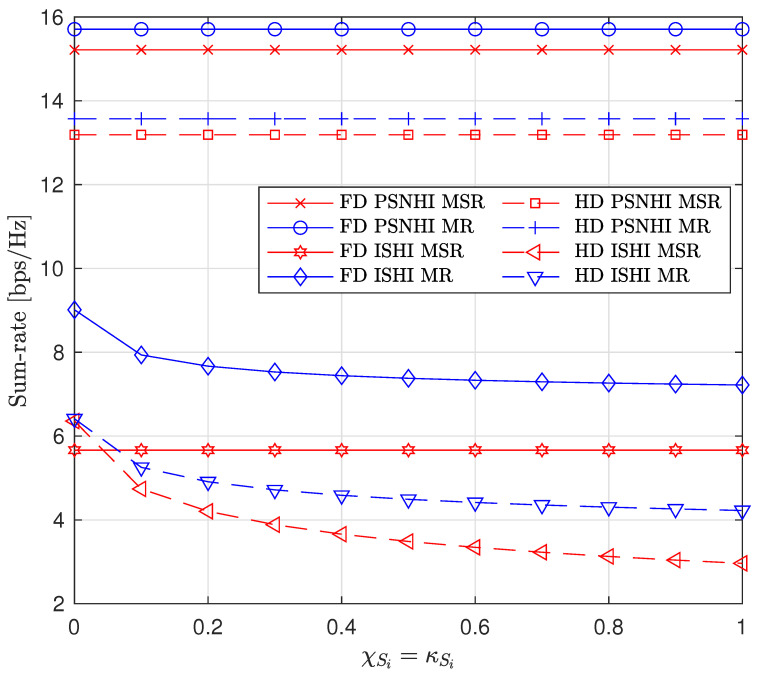
Sum rate versus imperfect cancellation coefficients ($\chi_{S_{c}}=\kappa_{S_{c}}$).

**Figure 7:**
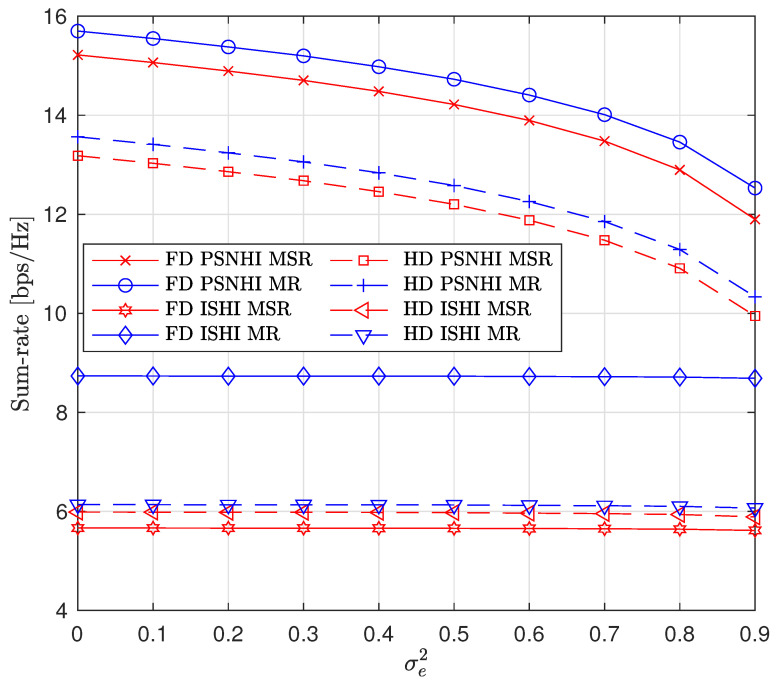
Sum rate versus CSI error variance ($\sigma_{e}^{2}$).

Next, we investigate the effects of imperfect cancellation (Imp-SIC and Imp-SuIC) and channel estimation error on the sum rate by varying the HI coefficients $\chi_{S_{i}}$, $\kappa_{S_{i}}$, and $\sigma_{e}^{2}$ (we assume that $\chi_{S_{i}}=\kappa_{S_{i}}$). First, the effect of varying $\chi_{S_{i}}$ and $\kappa_{S_{i}}$ is shown in Figure 6. In the PSNHI scenario, the sum-rate values are constant because the SIC and SuIC are perfect. In the ISHI scenario, the sum rate decreases with an increase in imperfection coefficients. However, the FD sum rate is constant under the MSR approach because of the STs’ insignificant rate values, and thus, the sum rate mainly consists of the $P_{2}$ rate and is not affected by either SIC or SuIC in this case. Second, the effect of imperfect CSI on the sum rate is shown in Figure 7. The small-scale channel model for imperfect CSI is $\zeta_{z_{1},z_{2}}={\hat{\zeta }}_{z_{1},z_{2}}+{\tilde{\zeta }}_{z_{1},z_{2}}$, where ${\hat{\zeta }}_{z_{1},z_{2}}∼\mathrm{CN}\left(0,1-\sigma_{e}^{2}\right)$ and ${\tilde{\zeta }}_{z_{1},z_{2}}∼\mathrm{CN}\left(0,\sigma_{e}^{2}\right)$ are the estimated and error channels, respectively, with a variance of $\sigma_{e}^{2}$ [22]. Under both the MR and MSR approaches, the sum rate significantly decreases with an increase in $\sigma_{e}^{2}$ in the PSNHI scenario. However, only a minute decrease in the sum-rate value is seen in the ISHI scenario due to the HIs present in the ISHI scenario, which already have a significant impact on the sum rate. Thus, the effect of $\sigma_{e}^{2}$ on top of the HIs is negligible.

**Figure 8:**
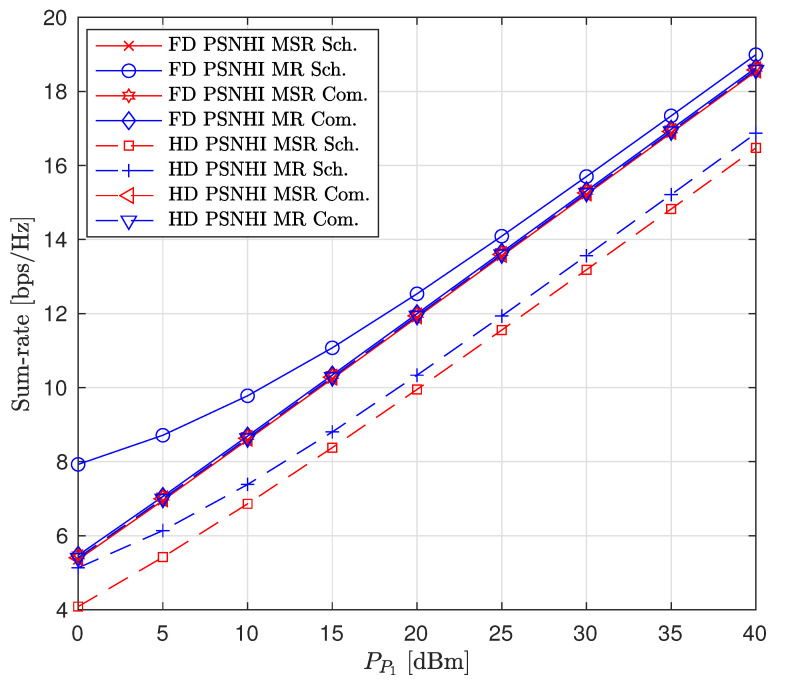
Sum rate versus primary transmitter (PT) transmit power ($P_{P_{1}}$).

**Figure 9:**
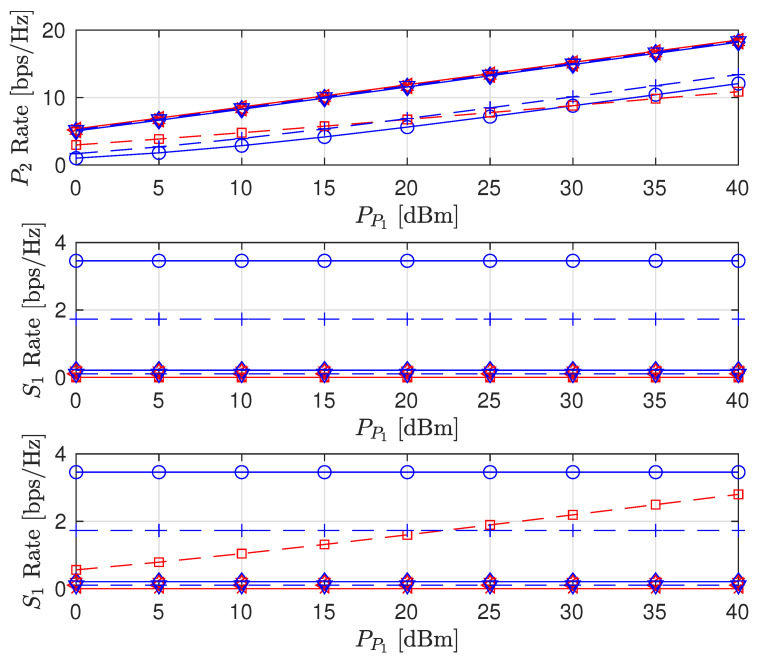
Achievable node rate versus PT transmit power.

**Figure 10:**
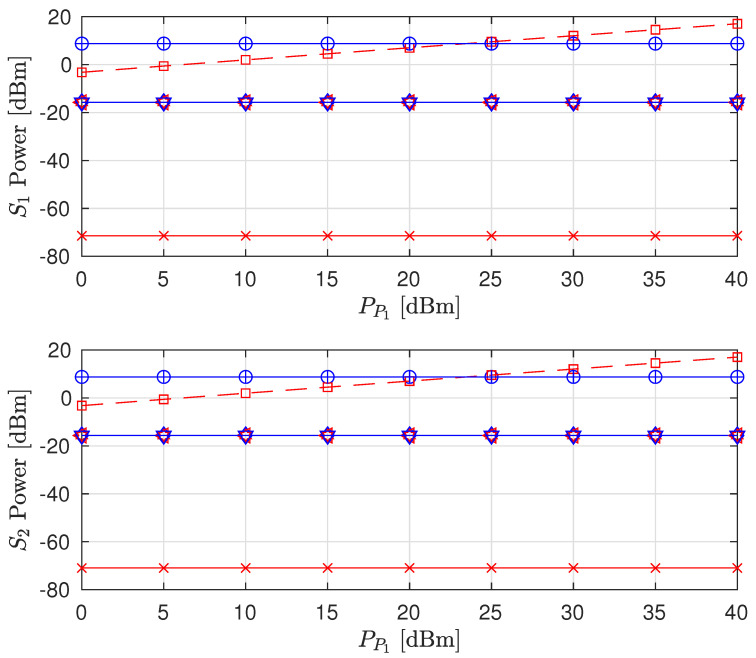
Secondary node transmit power versus PT transmit power.

A comparison of our proposed scheme (Sch) to the naive (Com) fixed interference power ($Q_{s}=-120$ dBm and β=0.5) [16] and fixed time allocation factor (α=0.5) for the PSNHI system structure are presented in Figure 8, Figure 9 and Figure 10. The MSR and MR sum-rates for both the Sch and Com improve with an increase in $P_{P_{1}}$, as shown in Figure 8. The increase in sum rates is mainly attributed to the rates achieved by the PL. In Figure 8, due to the constant $Q_{s}$ and α in the Com benchmark scheme, the MSR and MR have similar performances. However, the Com benchmark scheme underperforms the FD MR Sch. because the optimal interference power is determined in the FD MR Sch., producing better rates for the STs (shown in Figure 9), which improve the sum rate of the system. The MSR and MR sum rates for the Com benchmark scheme are suboptimal. However, their performances are similar to the FD MSR Sch because the FD MSR Sch approach achieves meager interference power (shown in Figure 10). Therefore, the FD MSR Sch approach obtains lower STs rates (Figure 9) and lower sum rates (Figure 8).

**Figure 11:**
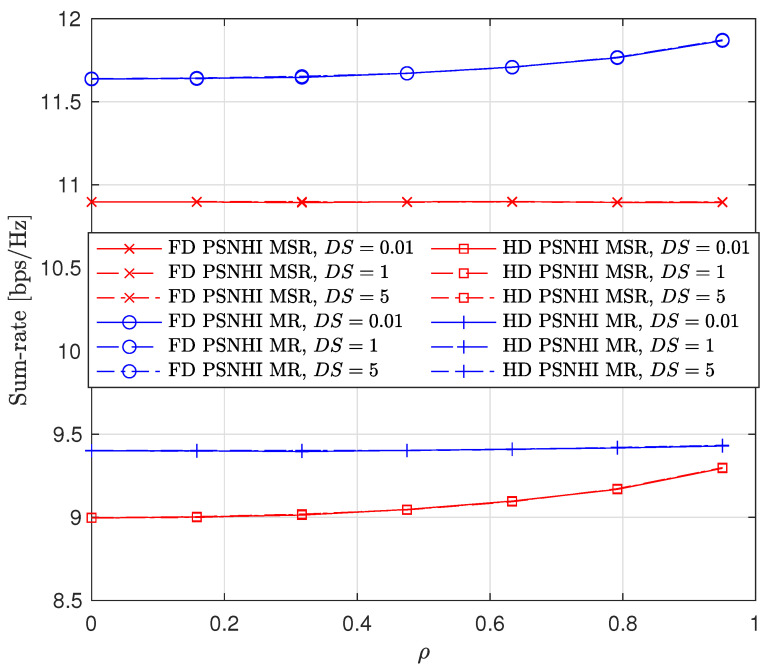
Sum rate versus covariance ρ where DS = {0.01,1,5} Hz.

**Figure 12:**
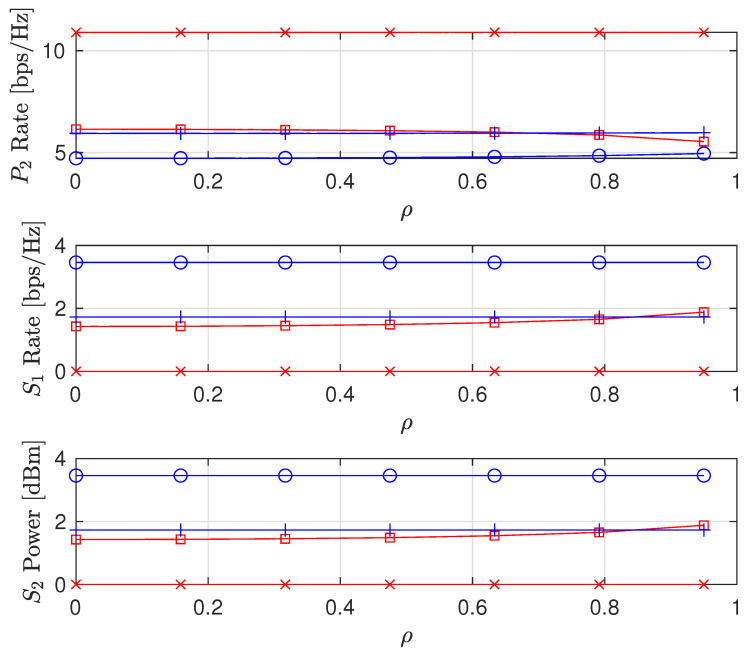
Achievable node rate versus covariance ρ where DS = {0.01,1,5} Hz.

**Figure 13:**
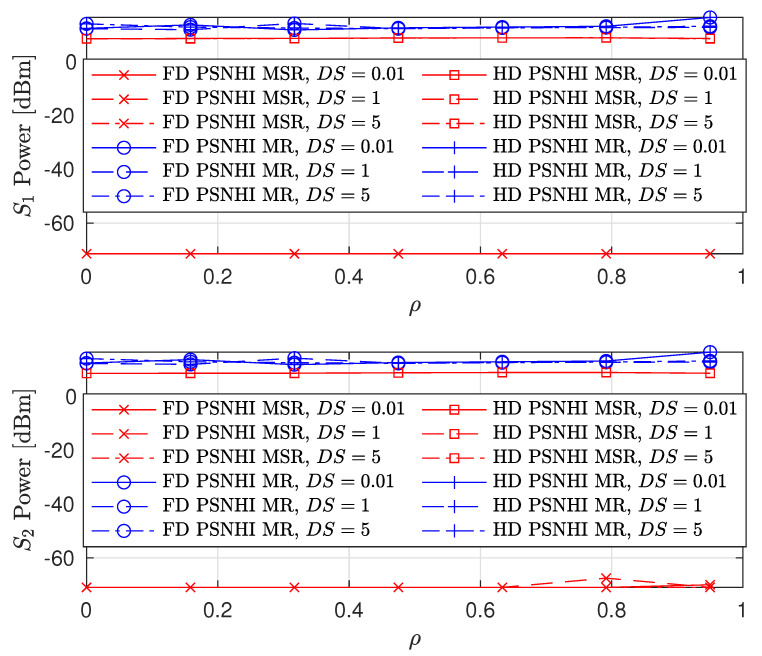
Secondary node transmit power versus covariance ρ where DS = {0.01,1,5} Hz.

We also consider the influence of time and spatial correlation in Rayleigh fading channels on sum-rate, PL and SL rates, and the STs transmit power as presented in Figure 11, Figure 12 and Figure 13, respectively. For time domain correlation, we consider various Doppler shift (DS) values. The spatial correlation of channels is parameterized by their covariance which we denote by ρ [31]. It can be observed from all the figures that the time correlation does not affect the performance of the algorithm. This is because the proposed algorithm makes a decision in a per-frame basis and we assume that a channel remains static during a frame time. However, the spatial correlation (increasing covariance) influences the performance of the algorithms and achieved values. The sum-rates of FD PSNHI MR and HD PSNHI MSR improve as the spatial correlation increases. Since more channels are involved in the FD system, both PL and SL benefit from spatial correlation higher in the FD system than the HD system. Hence, there is an improvement in the SL and PL rates achieved in the FD system compared to the HD system. FD MSR and FD MR have insignificant and significant increases, respectively, as shown in Figure 9. This causes a noticeable increase in the FD MR plot in Figure 11. With the HD system, STs’ transmission occur in two different time slots while PL transmission occurs in both time slots. Thus, there is a channel correlation between the PL and SL in each time slot. This implies that each ST has a better channel correlation with the PL system in a particular time slot. Therefore, the STs improve their rates compared to the PL, which shares two different correlated channels with two STs in two different time slots. This leads to the reduction in the PL rates for the MSR scheme, as shown in Figure 9. However, due to the rate constraints on the STs in the MR scheme, the PL can maintain its rate performance.

**Figure 14:**
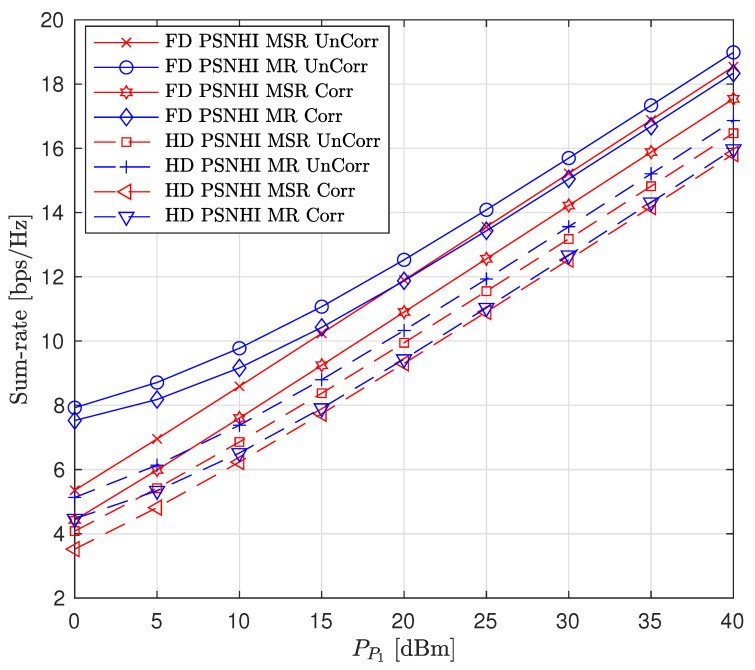
Sum rate versus primary transmitter (PT) transmit power ($P_{P_{1}}$) for correlated and uncorrelated Rayleigh fading channels where ρ=0.95 and DS = 5 Hz.

**Figure 15:**
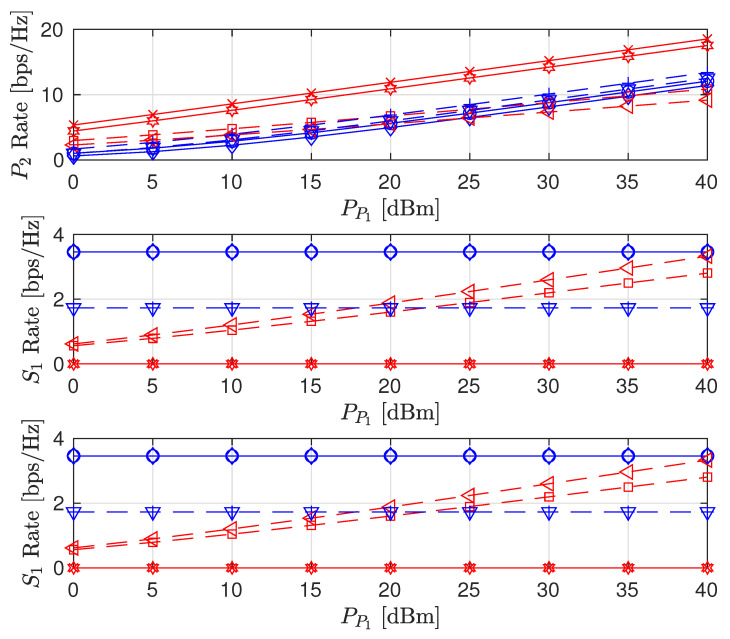
Achievable node rate versus PT transmit power for correlated and uncorrelated Rayleigh fading channels where ρ=0.95 and DS = 5 Hz.

**Figure 16:**
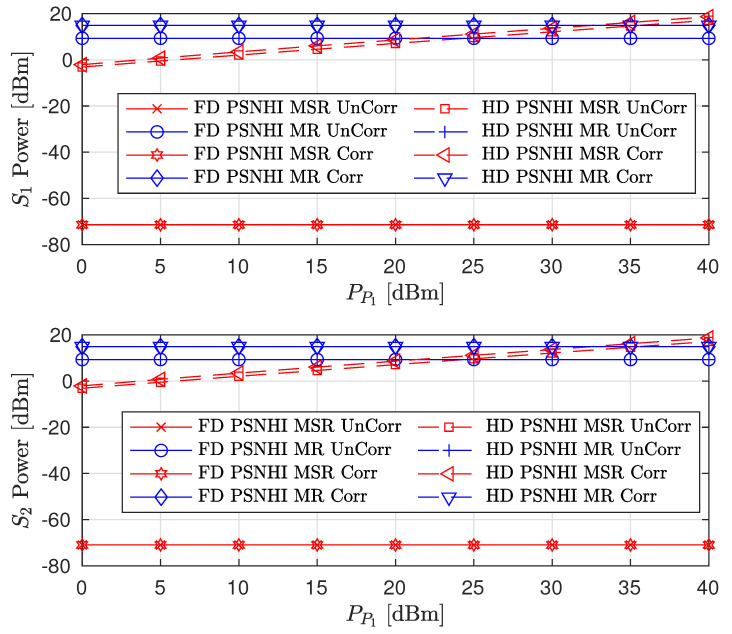
Secondary node transmit power versus PT transmit power for correlated and uncorrelated Rayleigh fading channels where ρ=0.95 and DS = 5 Hz.

Finally, the performance comparison of the proposed CRF between correlated (Corr) and uncorrelated (UnCorr) Rayleigh fading channels is discussed. For time domain correlation, we consider a channel sampling rate of 50 Hz and a Doppler shift (DS) of 5 Hz. For spatial correlation of channels, we set ρ to 0.95. The comparison results are given in Figure 14, Figure 15 and Figure 16. For the sum-rate plot in Figure 14, the UnCorr case performs better than the Corr channel case. This is because the Corr channels are a scaled version of the UnCorr channels by the correlation factor. This implies that the Corr has lower channel gains compared to the UnCorr channel if ρ<1. Therefore, in the Corr channel case, STs use higher transmit powers compared to the Uncorr channel case, as seen in Figure 16. Even though the Corr case transmits with higher power, it achieves the same rate values and constraints for the SL between Corr and Uncorr, as shown in Figure 15. However, the higher SL transmit power of the Corr case leads to higher interference power at the PL, leading to the Corr achieving a lower PL rate compared to the UnCorr case as presented in Figure 15. This behavior is transferred to the sum-rate plots in Figure 14 where the UnCorr case has better performance compared to the Corr case because of the PL rate performance difference.

## 7. Conclusions

This paper has investigated the power resource optimization problem for a CRF with a symbiotic BC-aided FD-based SL under various HI cases. In the proposed CRF, the SL consists of two FD nodes and both the PL and SL send information via a direct link and a BC-aided tag link. The problem was solved using two approaches: maximizing the sum rate and maximizing the PL rate subject to rate constraints on the SL. The solution for the FD mode was obtained with a simple root-finding algorithm, while that for the HD mode was derived in a closed form. The simulation results show that the sum rate and exploitation of the FD capabilities of the SL depend on both the problem objective and the HIs.

Extensions of this work can be the consideration of the maximization of the minimum rate of the system to identify further improvements in system performance. The replacement of the BC technology with intelligent reflective surfaces (IRS), multi-antenna systems, and interference at the secondary network from the PT are also potential extensions of the current work.

## Figures and Tables

**Table 1:** Simulation parameters.

Parameter	Symbol	Value	Unit
Network radius	−	30	m
Speed of light	$C_{L}$	$3\;\times 10^{8}$	m/s
Node carrier frequency	$f_{c}$	2.4	GHz
Bandwidth	BW	1	MHz
Transmitter antenna gain	$G_{T}$	6	dBi
Receiver antenna gain	$G_{R}$	6	dBi
Path-loss exponent	δ	3	-
Maximum transmit power	${\bar{P}}_{P_{1}}$, ${\bar{P}}_{S_{i}}$	30	dBm
Reflection coefficient	η	0.1	-
Attenuation coefficient	$A_{0}$	10	dB
Noise power	−	−175	dBm/Hz
Imp-SIC and Imp-SuIC coefficients	$\kappa_{S_{i}}$, $\chi_{S_{i}}$	0.01	-
Distortion noise variance	$\xi_{z}^{2}$	0.01	-
Time allocation factor	α	0.5	-
Total time resources	τ	1	s

## Data Availability

Not applicable.

## Abbreviations

The following abbreviations are used in this manuscript:

AWGN	Additive White Gaussian Noise
BC	Backscatter Communication
CR	Cognitive Radio
CRF	Cognitive Radio Framework
CSI	Channel State Information
FD	Full-Duplex
HD	Half-Duplex
HI	Hardware Impairments
Imp-SuIC	Imperfect Successive Interference Cancellation
Imp-SIC	Imperfect Self-Interference Cancellation
ISHI	Imperfect Self-interference Cancellation and Hardware Impairments
MR	Maximizing the primary link Rate
MSR	Maximizing the Sum Rate
SIC	Self-Interference Cancellation
SL	Secondary Link
ST	Secondary Transmitter
PL	Primary Link
PSNHI	Perfect Self-interference Cancellation and No Hardware Impairments
QoS	Quality of Service
